# Acute enteric-coated sodium bicarbonate has negligible effect on anaerobic performance but affects metabolomics and attenuates the gastrointestinal response

**DOI:** 10.3389/fphys.2022.996381

**Published:** 2022-10-13

**Authors:** Nihong Zhou, Yongzhao Fan, Xiangyu Wang, Junde Wang, Hao Wu

**Affiliations:** ^1^ Graduate School, Capital University of Physical Education and Sports, Beijing, China; ^2^ Qingdao Shengbang Health Food Co., Qingdao, China; ^3^ School of Kinesiology and Health, Capital University of Physical Education and Sports, Comprehensive Key Laboratory of Sports Ability Evaluation and Research of the General Administration of Sport of China, Beijing Key Laboratory of Sports Function Assessment and Technical Analysis, Beijing, China

**Keywords:** sodium bicarbonate supplementation, anaerobic performance, physiological profile, gastrointestinal reactions, metabolomics

## Abstract

Sodium bicarbonate ingestion before exercise has a performance-enhancing effect on high-intensity exercise. However, gastrointestinal symptoms can be a problematic side-effect. Enteric-coated sodium bicarbonate can attenuate gastrointestinal symptoms following acute bicarbonate loading. In addition, the subsequent effects on exercise performance and metabolomics have not been investigated. The purpose of this study was to investigate the acute effect of enteric-coated sodium bicarbonate supplementation on the anaerobic performance, physiological profile, and symptoms of gastrointestinal discomfort after severe-intensity intermittent exercise. At the same time, targeted metabolomics was used to study the changes in urine metabolism after ingestion of enteric-coated sodium bicarbonate and to explore the characteristics of biological metabolism. In a randomized crossover design, twelve male college students completed four Wingate anaerobic 30-s cycling tests (WACT) after consuming a placebo (PL) and two experimental conditions: 0.2 g/kg body mass in enteric-coated sodium bicarbonate pills (ES) or general sodium bicarbonate pills (GS). Blood lactate (BLA), heart rate (HR), ratings of perceived exertion (RPE), and gastrointestinal–symptoms assessment questionnaire (GSAQ) were measured pre-exercise and post-exercise. In contrast, mean power (MP) and peak power (PP) were recorded immediately post-exercise. Urine samples were collected before formal tests and 50 min after the third WACT. Our findings indicate the following: 1) mean power and peak power showed no significant difference among conditions (MP: F_2.0, 33_ = 0.541, *p* = 0.587, η^2^ = 0.032; PP: F_2.0, 33_ = 0.526, *p* = 0.596, η^2^ = 0.031). The PP decline of the ES and GS after the third WACT was lower than that of the PL; 2) There were no significant differences in physiological responses, such as BLA (F_2.0, 33.0 _= 0.191, *p* = 0.827, η^2^ = 0.011) and heart rate (F_2, 33_ = 0.418, *p* = 0.662, η^2^ = 0.025), between the three conditions. Although blood lactate concentration after 10 min of the third WACT was lower with ES and GS than with placebo; 3) Fewer participants experienced gastrointestinal symptoms with enteric-coated than with general sodium bicarbonate; 4) The metabolites with differences among the three conditions 50 min after exercise were 3-phospho-d-glycerate, d-Glucose 6-phosphate, pyruvate, cis-aconitate, oxaloacetate, and citrate. ES had higher levels of 3-phospho-d-glycerate, d-Glucose 6-phosphate, pyruvate, and cis-aconitate than GS. The 3-phospho-d-glycerate, d-Glucose 6-phosphate, pyruvate, and cis-aconitate levels in GS were significantly lower than in PL. In contrast, the citrate level in GS was significantly higher than that in other experimental conditions. Compared to PL, the level of oxaloacetate was higher after exercise in ES. This data suggests that supplementation of enteric-coated and general sodium bicarbonate before exercise can alter energy metabolism following anaerobic exercise, involving the metabolism of 3-phospho-d-glycerate, D-Glucose 6-phosphate, pyruvate, cis-aconitate, oxaloacetate, citrate, and lactate. However, they do not affect anaerobic performance and blood lactate. The supplementation of acute enteric-coated sodium bicarbonate and general sodium bicarbonate can enhance some of the weak effects of blood lactate clearance during anaerobic exercise, which may be beneficial for glycolytic energy supply. In addition, enteric-coated sodium bicarbonate intake mitigates gastrointestinal symptoms compared to general sodium bicarbonate.

## Introduction

During high-intensity exercise, the muscles mainly supplies energy through anaerobic metabolism to meet the energy demand (Gastin, 2001). In this process, it is inevitable to produce some metabolic acidic substances, such as lactate. The muscle lactate will cross the muscle cell membrane and enter the blood through diffusion or transfer. If the lactate cannot be removed in time, it will be decomposed into lactate and H^+^, resulting in H^+^ accumulation (Zinner et al., 2011). The increase of H^+^ will lead to an imbalance of acid-base balance and hinder the production of ATP (Juel, 2001), which will damage the contractile ability of muscles and ultimately affect exercise ability and performance. Therefore, it is very necessary to take reasonable measures to promote muscle acid-base balance during exercise.

Regulating the acid-base balance through nutrition is one of the important methods to alleviate fatigue and promote physical recovery (Naderi et al., 2016) quickly. During the past decades, numerous studies have demonstrated that increases in the extracellular buffer concentration *via* the oral ingestion of an alkaline solution, such as sodium bicarbonate may enhance human exercise performance (Wilkes et al., 1983; McNaughton et al., 1999; Requena et al., 2005; Lindh et al., 2008; Zabala et al., 2008; Ferreira et al., 2019). Sodium bicarbonate ingestion leads to an increase in plasma bicarbonate (HCO_3_^−^) concentration. The increase in extracellular pH leads to a greater transmembrane H+ concentration gradient, stimulating the co-transport of H+ and lactate from the exercising muscle cell. It has also been established that ingestion of Na^+^ can increase the plasma volume (Greenleaf and Brock, 1980), which could also benefit anaerobic activity by creating an enlarged buffering potential through dilution of the H^+^ concentration. Other studies have identified that acute or chronic exogenous HCO_3_^−^ may improve performance in 400 m races, high-intensity cycling, the Wingate test, and other anaerobic activities (Pouzash et al., 2012; Saunders et al., 2014; Dalle et al., 2021). Previous studies on the effect of sodium bicarbonate on anaerobic capacity were fewer. One study explored different levels of sodium bicarbonate intake that influenced acid-base balance and performance during high-intensity exercise (DouroudosFatouros et al., 2006), and another study examined the effect of sodium bicarbonate ingestion on performance and perceptual responses (Zabala et al., 2008).

Based on a comprehensive review of the international Society of Sports Nutrition position on sodium bicarbonate and exercise performance, the ergogenic effects of sodium bicarbonate are mostly established for exercise tasks of high-intensity that last between 30 s and 12 min (Driller et al., 2012; Mündel, 2018; Grgic et al., 2021; Grgic, 2022). The Wingate test is a common method to evaluate anaerobic exercise performance and has been used more often in studies related to sodium bicarbonate. However, the results of exploring the performance of sodium bicarbonate on the Wingate test were found to be ambiguous (Zabala et al., 2008; Zabala et al., 2011; Saunders et al., 2014). Based on previous literature (Zinner et al., 2011), the present study adapted the exercise protocol and used four Wingate anaerobic 30s cycling tests as the exercise protocol. Sodium bicarbonate supplementation (doses from 0.2 to 0.5 g/kg) may improve the performance of muscular endurance activities. Although 0.2 g/kg of sodium bicarbonate appears to be the minimum dose needed to improve exercise performance, there are still findings of gastrointestinal discomfort at this dose (Gough et al., 2017). Based on the literature and pre-experimental results, an intake dose of 0.2 g/kg was used in this study.

Sodium bicarbonate supplementation has the possibility of gastrointestinal discomfort, resulting in symptoms such as nausea, stomach pain, diarrhea, and vomiting (Burke and Pyne, 2007). One study also discovered that abdominal distress was significantly more prominent in the sodium bicarbonate trial than the placebo, resulting in an increase in stomach cramping, stomach ache, and diarrhea in the participants immediately after consumption. A larger increase in symptoms may have impacted performance (Miller et al., 2016). By applying a novel ingestion strategy to an enteric coating (containing hydroxypropyl methylcellulose, shellac, *etc.*), it resists dissolution in the gastric acid environment, and acid-sensitive components such as sodium bicarbonate can bypass the gastric (Barbosa et al., 2017). Since GI distress during sodium bicarbonate application may be partly attributable to degradation in the stomach (Turnberg et al., 1970), enteric coating can reduce the neutralizing effect of gastric acid and largely reduce side effects. Some studies have reported that enteric-coated sodium bicarbonate can attenuate gastrointestinal symptoms following acute bicarbonate loading on aerobic exercise performance have been investigated (Hilton et al., 2020). Evidence suggests that athletes may be deterred from supplementing with sodium bicarbonate due to the risk of GI symptoms during training or competition (Heibel et al., 2018). However, fewer studies have been conducted on the effects of acute enteric-coated sodium bicarbonate supplementation on the physiological profile, anaerobic performance, and symptoms of gastrointestinal discomfort.

Metabolomics is a new approach after genomics, proteomics, and transcriptomics with the advantages of high throughput, high specificity, and high sensitivity. Metabolomics is routinely applied as a tool for biomarker discovery to understand the systems-level effects of metabolites (Johnson et al., 2016). It is the profiling of metabolites in biofluids, cells, and tissues. The use of urine provides an ideal non-invasive method that results in a large overview of the metabolite matrix (Daskalaki et al., 2015). With the recent emergence of metabolomics analyses, metabolomics is now being applied to choosing exercise and nutritional supplements (Nieman et al., 2015; Al-Khelaifi et al., 2018). Metabolomics analysis can be either targeted (that focused on quantitative measurements of usually small numbers of metabolites) or untargeted (that focused on metabolic profiling of the total complement of metabolites for the studied samples) (Emwas et al., 2021). Targeted metabolomics focuses on analyzing several selected metabolites, such as studies related to specific metabolic pathways, drug toxicology, and specific effects of certain foods/nutrients. However, metabolomics analysis methods are less applied in the study of enteric-coated sodium bicarbonate.

Some studies have examined sodium bicarbonate supplementation during high-intensity exercise affects performance. However, the findings are equivocal, and some studies did not find significant differences between sodium bicarbonate and placebo conditions (Joyce et al., 2012; Driller et al., 2013). The researchers hypothesized that the finding may have been due to the unique subject characteristics (highly trained members of national teams) (Edge et al., 2004; Carr et al., 2011; Wang et al., 2019). Based on these findings, healthy college students were chosen to be the participants of this study instead of highly trained athletes, to better understand the possible impact of sodium bicarbonate ingestion when coupled with high-intensity exercise.

The results of past studies led us to develop a research project that would investigate the effects of acute enteric-coated sodium bicarbonate supplementation on anaerobic performance, heart rate, blood lactate, and symptoms of gastrointestinal discomfort in healthy young men. The target metabolomics based on the multi-reaction monitoring technology (MRM) method was used to analyze the specific metabolic pathway. It was hypothesized that both supplementation conditions would improve anaerobic performance. However, enteric-coated sodium bicarbonate was more effective in reducing the incidence of gastrointestinal side effects.

## Materials and methods

### Participants

Fifteen healthy, college-aged men participated in the study (but three participants failed to complete the entire trial, n=12). The basic information of the participants is presented in Table 1. Participants were excluded from the study if they were smokers, taking medication, drinking any beverages other than water, or having any chronic diseases. No participants reported taking any performance-enhancing supplements or supplements that would be classified as either intracellular or extracellular buffers. Participants were asked to refrain from maximal exercise 48 h before each trial, and each subject was advised to maintain a consistent diet and activities. All participants were informed of both the benefits and the potential side effects of the study (both verbally and in writing) before providing written informed consent. The institutional research ethics committee of Capital University of Physical Education and Sports granted ethical permission (2021A42).

**TABLE 1:** Basic information of participants (mean ± SD).

Sex	Number	Age (Years)	Height (cm)	Weight (kg)	Bmi
Male	12	24.25 ± 0.75	179.08 ± 2.42	74.43 ± 6.12	23.19 ± 1.60

### Procedures

**FIGURE 1:**
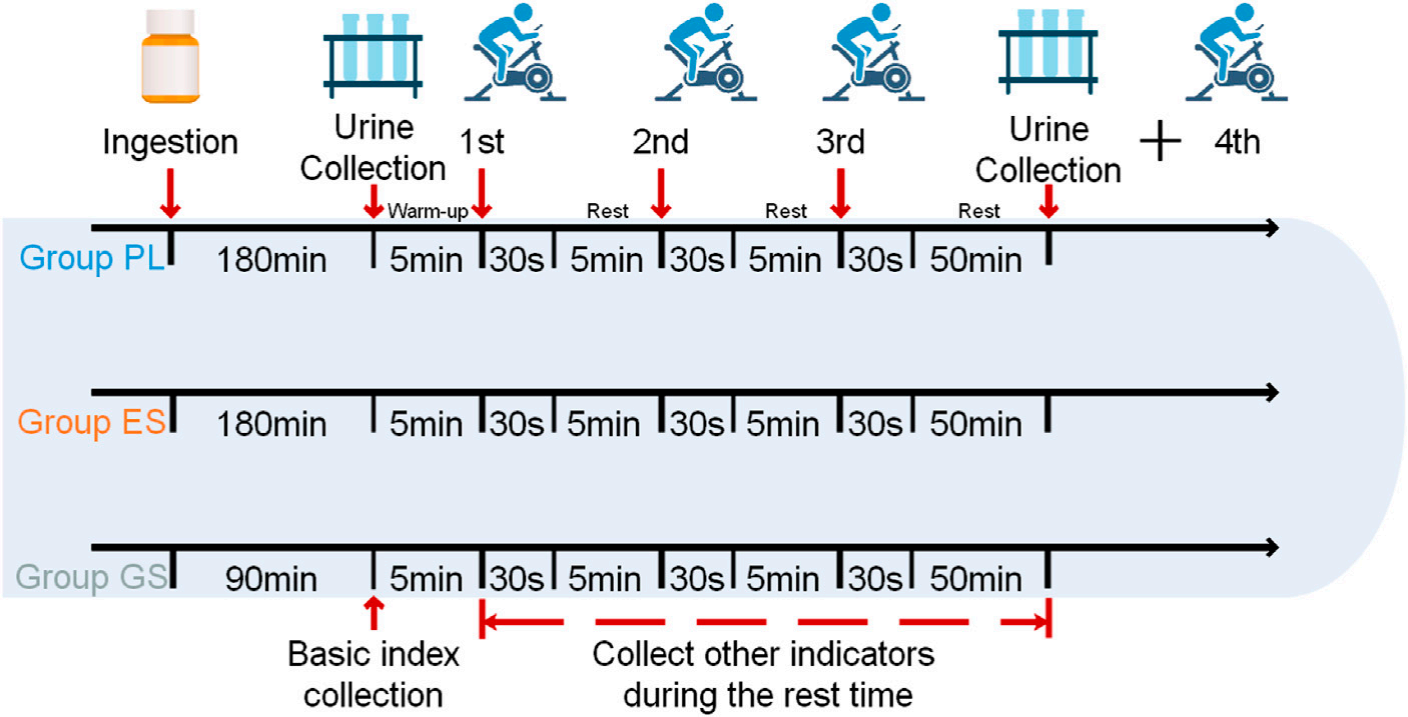
Main procedure of the trials. placebo (PL), enteric-coated sodium bicarbonate (ES), general sodium bicarbonate (GS). 1st: the first WACT; second: the second WACT; third: the third WACT; fourth: the fourth WACT.

The study was divided into three trials including enteric-coated sodium bicarbonate (ES, containing enteric coating and sodium bicarbonate), general sodium bicarbonate (GS, containing sodium bicarbonate), and placebo (PL, containing cornstarch), and was conducted in a randomized crossover design. The supplementation dose was 0.2 g/kg body mass in the form of unmarked oval tablets for each group of experiments. The recommended timing of sodium bicarbonate intake was 90–180 min prior to exercise or competition (Jones et al., 1977; Bishop et al., 2004; Yuen, 2010; Grgic et al., 2021). Enteric-coated sodium bicarbonate is chosen to be ingested 180 min before exercise due to the additional time required for digestion and absorption in the intestine (Maderuelo et al., 2019). ES and PL were ingested 180 min prior to testing, and GS was ingested 90 min prior to testing. Each experiment was separated by at least 7 days (1 week was considered a sufficient washout period to eliminate any ergogenic effect of sodium bicarbonate (Bishop and Claudius, 2005)). All trials were performed at the same time of day to minimize diurnal variation. The Wingate anaerobic 30-s cycling test (WACT, resistance factor was set at 0.075 kg/kg BW) was used in this study. Prior to the test, participants adjusted handlebar and seat position (same position in trials), and then participants performed a standardized warm-up (power: 60 W; time: 5 min). Participants performed the first three WACTs to test anaerobic capacity with a 5-min interval each time (passive recovery, trying to remain seated; small amounts of water allowed, totaling 200 ml). This was followed by a third interval of 50 min (passive recovery; no water or other food intake to avoid interfering with urine sampling), and a fourth WACT was performed to test recovery of anaerobic capacity. Throughout the test, participants were given verbal encouragement and asked to complete the WACTs as quickly as possible. The specific experimental procedure is shown in Figure 1.

### Experimental design

Upon arrival at the laboratory, participants were asked to ingest ES, GS, or PL for the next 30 min. At a specified time after the start of supplement intake (ES and PL: 180 min, GS: 90 min), participants started performing four Wingate anaerobic 30-s cycling tests with a Monark 894E (Ergomedic 894E, Sweden) after a baseline (pre-trial) capillary blood sample was taken. Samples were taken at the start, in the middle, and at the end of WACT. The participants had the index collection to establish basal measurements (blood lactate (BLA), heart rate (HR), ratings of perceived exertion (RPE), gastrointestinal-symptoms assessment questionnaire (GSAQ), and urine) before the first WACT. Capillary blood samples to determine BLA concentration were collected from the ear lobes *via* the h/p/cosmos Sirius® lactate test meter (manufacturer: SensLab GmbH, Germany) at the third minute of the five-minute interval after the first three WACT. In the third WACT, measurements of BLA were conducted immediately at 1, 4, 7, 10, 15, 20, 25, and 30 min after the cessation of exercise. The HR was monitored by the Polar Rs 800cx telemetry heart rate monitor (manufacturer: Polar, Finland) from the resting state to the recovery period for analyzing the heart rate. RPE was measured with the Borg 15-point scale, ranging from 6 (very, very light) to 20 (very, very heavy).

HR and RPE were measured immediately after the first three WACTs and the end of the third WACT, as well as 1, 4, 7, 10, 15, 20, 25, and 30 min after the third WACT. Due to the gastrointestinal side effects associated with sodium bicarbonate ingestion (Carr et al., 2012), participants completed a GSAQ before the test. This was followed by collecting for GSAQ (immediately after the third WACT and before the fourth WACT). The numerical rating scale (NRS) (scale 0–10, with zero reflecting no gastrointestinal discomfort and 10 indicating the most severe gastrointestinal discomfort) was used to classify the intensity of these symptoms (Dworkin et al., 2005). After that, the fourth WACT was conducted. The mean power (MP) and peak power (PP) of the test were calculated *via* Monark Anaerobic Testing software after every WACT. Urine was collected before warm-up and 50 min after the third WACT.

### Urine metabolomics

The metabolomics in this study is a targeted metabolomics analysis based on the MRM approach. This detection method covers important metabolites in the tricarboxylic acid cycle, the glycolytic pathway, and oxidative phosphorylation processes. The MRM principle uses selective response/multi-reaction monitoring technology (SRM/MRM) to detect and analyze specific metabolites in standard samples. Absolute quantitative results of the target metabolites can be obtained, which have the characteristics of high specificity, high sensitivity, and high accuracy.

The samples were removed from -80°C, slowly dissolved at 4°C, and 1000ul of pre-cooled methanol acetonitrile solution (1:1, v/v) was added to each group of samples, Vortex for 60 s, -20°C for 1 h to precipitate the protein, 14000rcf, centrifuge at 4°C for 20 min, freeze dry the supernatant, -80°C to store the samples.

The samples were separated using an Agilent 1290 Infinity LC Ultra-Performance Liquid Chromatography system. The mobile phase contained A = 10 mM aqueous ammonium acetate solution and B = acetonitrile. The samples were in the automatic sampler at 4°C, and the column temperatures were kept constant at 45°C, The gradients were at a flow rate of 300 μl/min, a 2 µl aliquot of each sample was injected. The relevant liquid phase gradients were as follows: the gradient was 90% B linearly reduced to 40% in 0–18 min, and then added to 90% in 0.1 min, and then maintained at 90% from 18.1 to 23 min. The sample cohort is set up with one QC sample for each interval of a certain number of experimental samples, which is used to detect and evaluate the stability and reproducibility of the system. The sample cohort is set up with a mixture of standards of energy metabolites for chromatographic retention time correction.

Mass spectrometry was performed using a 5500 QTRAP mass spectrometer (AB SCIEX) in negative ion mode. The 5500 QTRAP ESI source conditions are as follows: source temperature 450°C, ion Source Gas1 (Gas1): 45, Ion Source Gas2 (Gas2): 45, Curtain gas (CUR): 30, ionSapary Voltage Floating (ISVF)-4500 V; Adopt the MRM mode detection ion pair.

## Statistical analysis

Data were entered into Microsoft Excel 2010 and analyzed with SPSS 26.0. Data were analyzed using parametric tests following confirmation of a normal distribution *via* the Shapiro-Wilks-W-test and are presented as mean ± standard deviation (SD). Normally distributed data sets were analyzed with appropriate parametric statistical tests. Repeated-measures ANOVA compared conditions for significant differences among the three trials (MP, PP, BLA, HR, and RPE). Simple effect analysis was performed for the horizontal comparison of between-group factors when there was an interaction, and longitudinal comparison of within-group factors was performed when there was no interaction. In order to evaluate differences following the ingestion of GS and ES, effect sizes were calculated for each variable. In all cases, the significance level was set at *P* < 0.05. In addition, urine sample data was extracted by Multiquant software for peak area and retention time. The retention time was corrected using standards of energy metabolites for metabolite identification.

## Results

### Wingate anaerobic 30s cycling test

**FIGURE 2:**
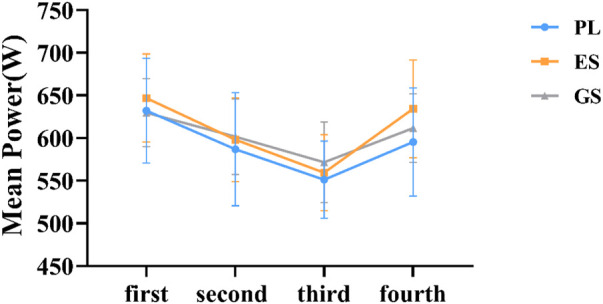
Trend of mean power. The horizontal coordinate represents the order of WACT in the experiment. The figure represents the mean ± SD of mean power data for three conditions.

**FIGURE 3:**
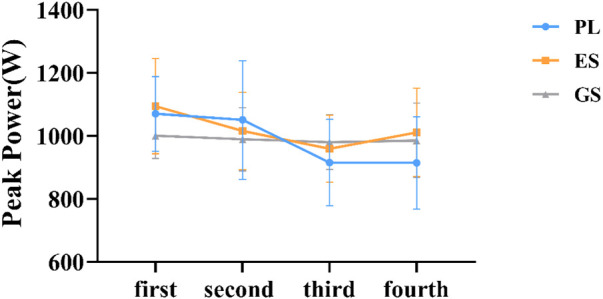
Trend of peak power. The horizontal coordinate represents the order of WACT in the experiment. The figure represents the mean ± SD of peak power data for three conditions.

An overall main effect for WACT test was not apparent (*p* > 0.05) (Figures 2, 3). A comparison of the trials indicated no differences in MP completed among the three conditions of trials (MP: F_2.0, 33_ = 0.541, *p* = 0.587, η^2^ = 0.032; PP: F_2.0, 33_ = 0.526, *p* = 0.596, η^2^ = 0.031). There was a significant main effect of time on MP (F_2.867, 94.606_ = 37.103, *p* = 0.000, η^2^ = 0.529) and PP (F_2.821, 93.108_ = 6.929, *p* = 0.000, η^2^ = 0.174), but no interaction effect between condition and time (MP: F_5.734, 94.606_ = 1.166, *p* = 0.332, η^2^ = 0.066; PP: F_5.643, 93.108_ = 1.973, *p* = 0.082, η^2^ = 0.107). However, ingestion of ES and GS slightly increased MP compared with PL in the three experimental conditions (The fourth WACT MP: ES = 634.36 ± 57.21W, GS = 611.74 ± 40.32W, PL = 595.40 ± 63.37W; *p* > 0.05), despite no significant differences being observed in all the performance variables analyzed between successive tests (Figure 2). The PP of the ES and GS decreased slower than that of the PL from the second WACT (The fourth WACT PP: ES = 1011.31 ± 139.76 W, GS = 985.18 ± 118.40W, PL = 914.53 ± 146.75W; *p* > 0.05), based on the changing trend of four peak powers (Figure 3).

### Blood lactate

**FIGURE 4:**
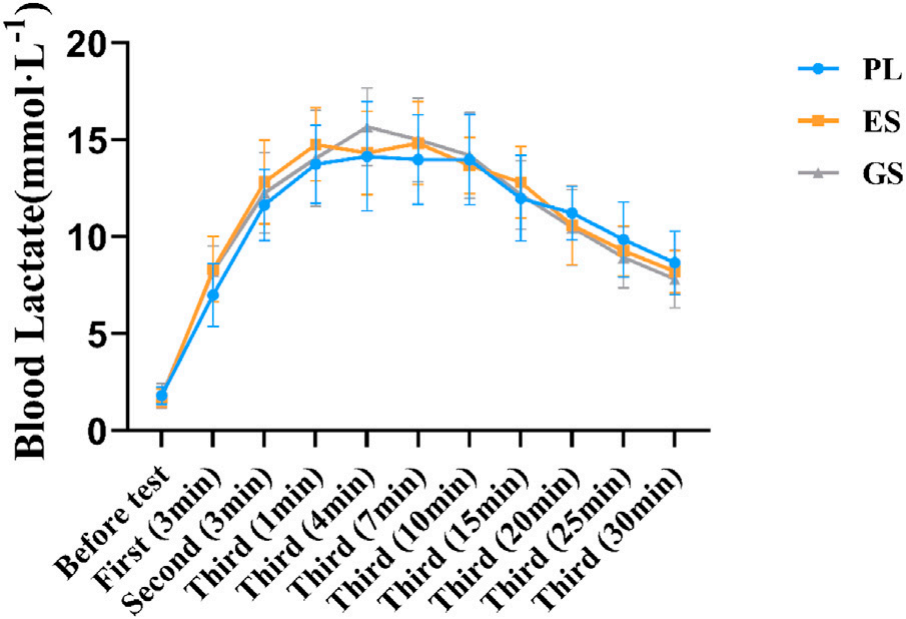
Blood lactate responses during the experiments. Values are means ± SD. First (n min): n minutes after the first WACT; Second (n min): n minutes after the second WACT; Third (n min): n minutes after the third WACT.

In the three experimental conditions, there was no difference in the resting BLA, either in the first two WACT or other periods of the third WACT (see Figure 4).

ES and GS ingestion did not alter BLA (F_2.0, 33.0 _= 0.191, *p* = 0.827, η^2^ = 0.011) despite significant changes post-exercise (F_5.971, 197.037_ = 289.572, *p* = 0.000, η^2^ = 0.898), with no condition × time interaction (F_11.942, 197.037_ = 1.703, *p* = 0.069, η^2^ = 0.094). However, at 7 min after the third WACT under experimental conditions, BLA values were higher in ES and GS than in PL (ES = 14.83 ± 2.13 mmol L^−1^, GS = 15.00 ± 2.16 mmol L^−1^, PL = 13.98 ± 2.30 mmol L^−1^; *p* > 0.05). The results of all three experiments showed that the BLA of the ES and GS was lower than that of the PL on the descent after 10 min of the third WACT (After 20 min of the third WACT: ES = 10.59 ± 2.03 mmol L^−1^, GS = 10.48 ± 1.96 mmol L^−1^, PL = 11.22 ± 1.39 mmol L^−1^; *p* > 0.05). From the downward trend in Figure 4, the BLA of the ES and GS decreased more than the PL.

### Heart rate

The HR was increasing with time during the whole experiment (F_4.847, 159.967_ = 581.113, *p* = 0.000, η^2^ = 0.946). However, no significant differences were found between conditions (F_2, 33_ = 0.418, *p* = 0.662, η^2^ = 0.025), and there was no significant condition × time interaction (F_9.695, 159.967_ = 0.565, *p* = 0.836, η^2^ = 0.033).

Enteric-coated sodium bicarbonate consumption had no influence on HR (e.g., before the test: ES = 79.25 ± 9.07, GS = 73.25 ± 8.94, PL = 80.25 ± 8.18; at 30 min after the third exercise: ES = 100.58 ± 7.79, GS = 97.08 ± 12.35, PL = 101.17 ± 8.99; *p* > 0.05). As with GS, there was no difference between the three experimental conditions at these moments during the four WACTs (Table 2, all *p* > 0.05).

**TABLE 2:** HR responses during three experimental conditions.

Time	PL (beats/minute)	ES (beats/minute)	GS (beats/minute)
Before test	80.25 ± 8.18	79.25 ± 9.07	73.25 ± 8.94
First (Immediately)	165.42 ± 18.84	167.50 ± 13.33	166.08 ± 16.64
First (4min)	114.42 ± 16.26	111.08 ± 10.11	105.75 ± 13.53
Second (Immediately)	175.58 ± 13.75	172.33 ± 11.74	171.00 ± 13.76
Second (4min)	128.33 ± 13.55	127.00 ± 14.89	121.25 ± 15.33
Third (Immediately)	174.17 ± 13.66	173.92 ± 12.48	171.08 ± 13.87
Third (1min)	148.25 ± 18.76	150.42 ± 13.67	145.50 ± 15.53
Third (4min)	123.50 ± 14.49	125.50 ± 14.43	119.08 ± 14.34
Third (7min)	115.67 ± 9.99	115.92 ± 11.82	113.08 ± 15.02
Third (10min)	112.83 ± 14.57	109.75 ± 11.31	108.17 ± 11.01
Third (15min)	106.17 ± 9.86	107.50 ± 10.56	107.33 ± 15.28
Third (20min)	105.17 ± 9.65	105.58 ± 11.05	103.75 ± 13.82
Third (25min)	101.92 ± 7.46	102.17 ± 10.02	99.42 ± 12.59
Third (30min)	101.17 ± 8.99	100.58 ± 7.79	97.08 ± 12.35

Values are means ± SD. First (n min): n minutes after the first WACT; Second (n min): n minutes after the second WACT; Third (n min): n minutes after the third WACT.

### Gastrointestinal–symptoms assessment questionnaire

Among all three conditions, the greatest incidence of GI side effects was recorded among the GS group. Overall, two participants in the ES and two participants in the PL reported mild side effects. However, seven participants suffered from obvious side effects during the GS study. Side effects emerged after general sodium bicarbonate supplementation, most often immediately after the third WACT.

**FIGURE 5:**
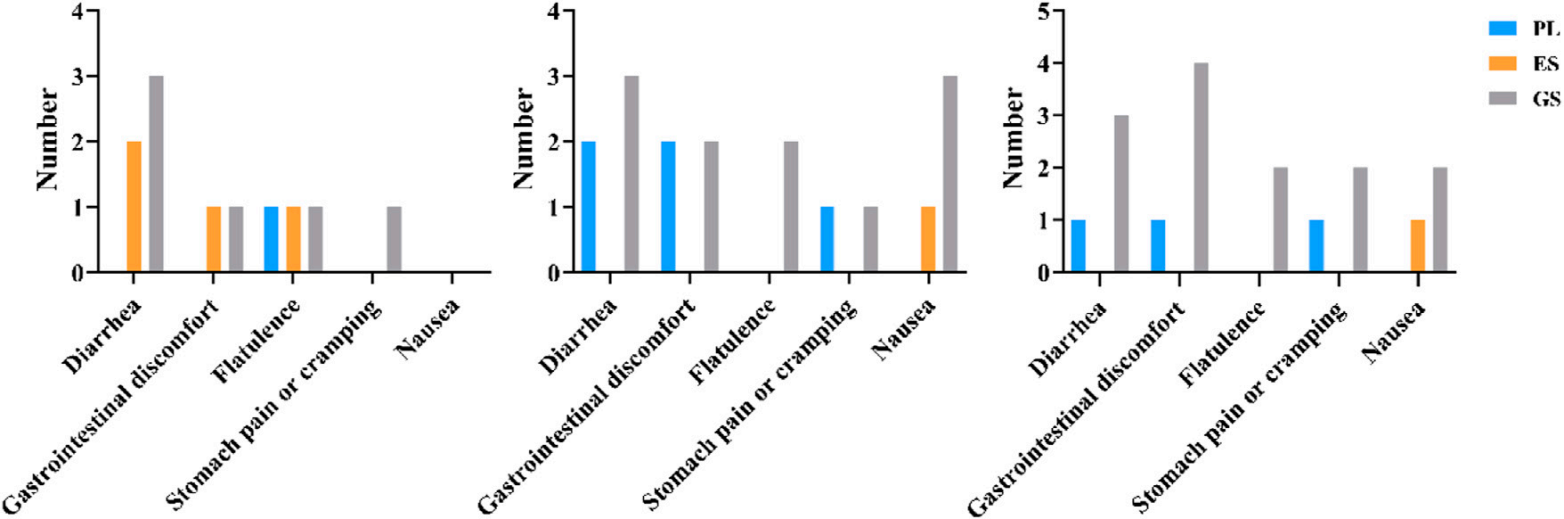
Gastrointestinal–symptoms assessment questionnaire results before exercise, immediately, and 50 min after the third WACT.

Figure 5 indicates the main symptoms of gastrointestinal reactions before exercise, immediately after the third WACT, and 50 min after the third WACT. Analysis of the circumstances of acute GI discomfort in the GS indicated more cases of diarrhea, flatulence, gastrointestinal discomfort, and nausea. Figure 5 shows a significant increase in all of the parameters measured at 90 min after general sodium bicarbonate ingestion. However, the ES had no obvious gastrointestinal tract symptoms after exercise.

### Ratings of perceived exertion

There were no differences in either RPE (F_2.0, 33.0_ = 0.362, *p* = 0.699, η^2^ = 0.021) between conditions (Table 3), although there were significant increases in RPE during the experiment (F_3.552, 117.209_ = 143.397, *p* = 0.000, η^2^ = 0.813). No significant condition × time interactions were revealed for neither RPE (F_7.104, 117.209_ = 0.628, *p* = 0.734, η^2 ^= 0.037).

**TABLE 3:** RPE in three conditions at each moment.

Time	PL	ES	GS
Before test	8.33 ± 1.67	8.00 ± 1.86	8.58 ± 1.31
First (Immediately)	14.33 ± 1.50	13.58 ± 1.31	13.92 ± 1.84
First (4min)	12.33 ± 1.67	11.42 ± 1.78	12.33 ± 1.50
Second (Immediately)	15.33 ± 1.56	15.00 ± 1.48	15.33 ± 1.15
Second (4min)	13.50 ± 1.62	13.17 ± 2.08	13.67 ± 1.50
Third (Immediately)	15.92 ± 1.44	16.08 ± 1.62	16.42 ± 1.56
Third (1min)	15.58 ± 1.00	15.75 ± 1.66	15.83 ± 1.59
Third (4min)	14.08 ± 1.51	14.00 ± 2.09	14.42 ± 1.78
Third (7min)	12.58 ± 1.62	12.92 ± 1.78	13.50 ± 1.51
Third (10min)	11.67 ± 1.67	11.92 ± 1.88	12.50 ± 1.93
Third (15min)	11.00 ± 1.71	11.50 ± 1.68	11.58 ± 2.15
Third (20min)	10.83 ± 1.27	11.08 ± 1.62	11.42 ± 2.27
Third (25min)	10.58 ± 1.24	11.08 ± 1.93	11.25 ± 2.26
Third (30min)	10.50 ± 1.31	10.58 ± 1.98	11.17 ± 2.44

Values are means ± SD. First (n min): n minutes after the first WACT; Second (n min): n minutes after the second WACT; Third (n min): n minutes after the third WACT.

### Urine metabolomic analyses

**FIGURE 6:**
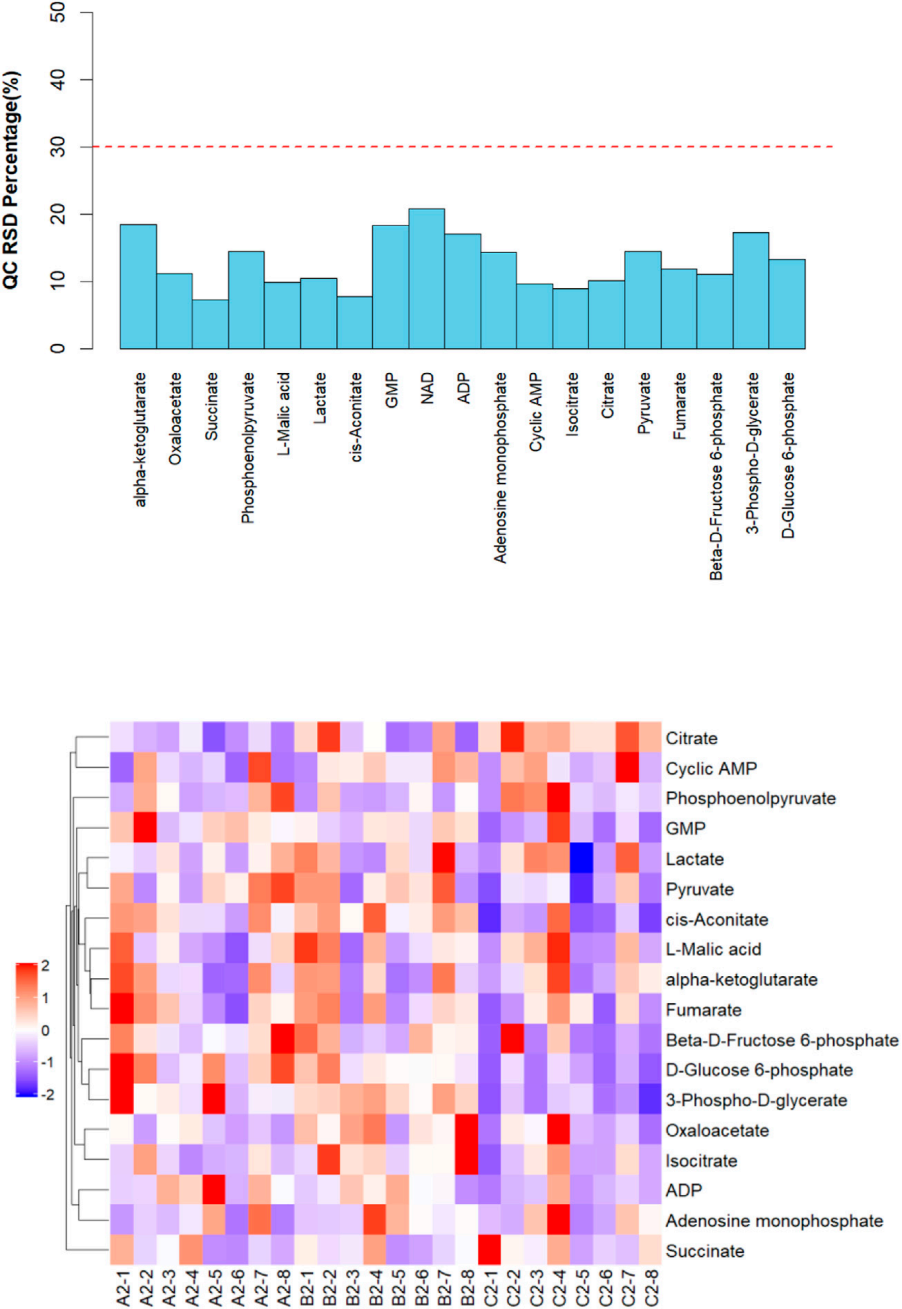
RSD distribution of QC samples, and urine metabolite hierarchy clustering plots post-test.

The targeted metabolomics tests cover important metabolites in the tricarboxylic acid cycle, glycolytic processes, and oxidative phosphorylation processes. Eight samples were screened from each group of urine samples for metabolomics analysis. QC samples were prepared by mixing all samples in equal quantities, and the QC samples were used to evaluate the stability and reproducibility of the data. The RSD (relative standard deviation) results of the measured substances in the QC samples are presented in Figure 6, where the energy metabolism with RSD is less than 30%, indicating that the data in the samples were stable and reliable. The metabolite hierarchy clustering plots are presented in Figure 6. When the candidate metabolites are screened reasonably well and accurately, the same condition of samples can appear in the same cluster through clustering. At the same time, metabolites clustered in the same cluster have similar expression patterns and may be in closer reaction steps in the metabolic process.

After testing and analysis, eighteen metabolites were observed in this study. A total of five differential metabolites were screened 50 min after the experiment with the three conditions for differences in urinary metabolites, namely 3-phospho-d-glycerate, d-Glucose 6-phosphate, pyruvate, cis-aconitate, oxaloacetate, and citrate. Screening analysis of pre-exercise after supplementation found that the level of lactate was significantly higher in ES than in PL. However, there was no significant difference after exercise.

**FIGURE 7:**
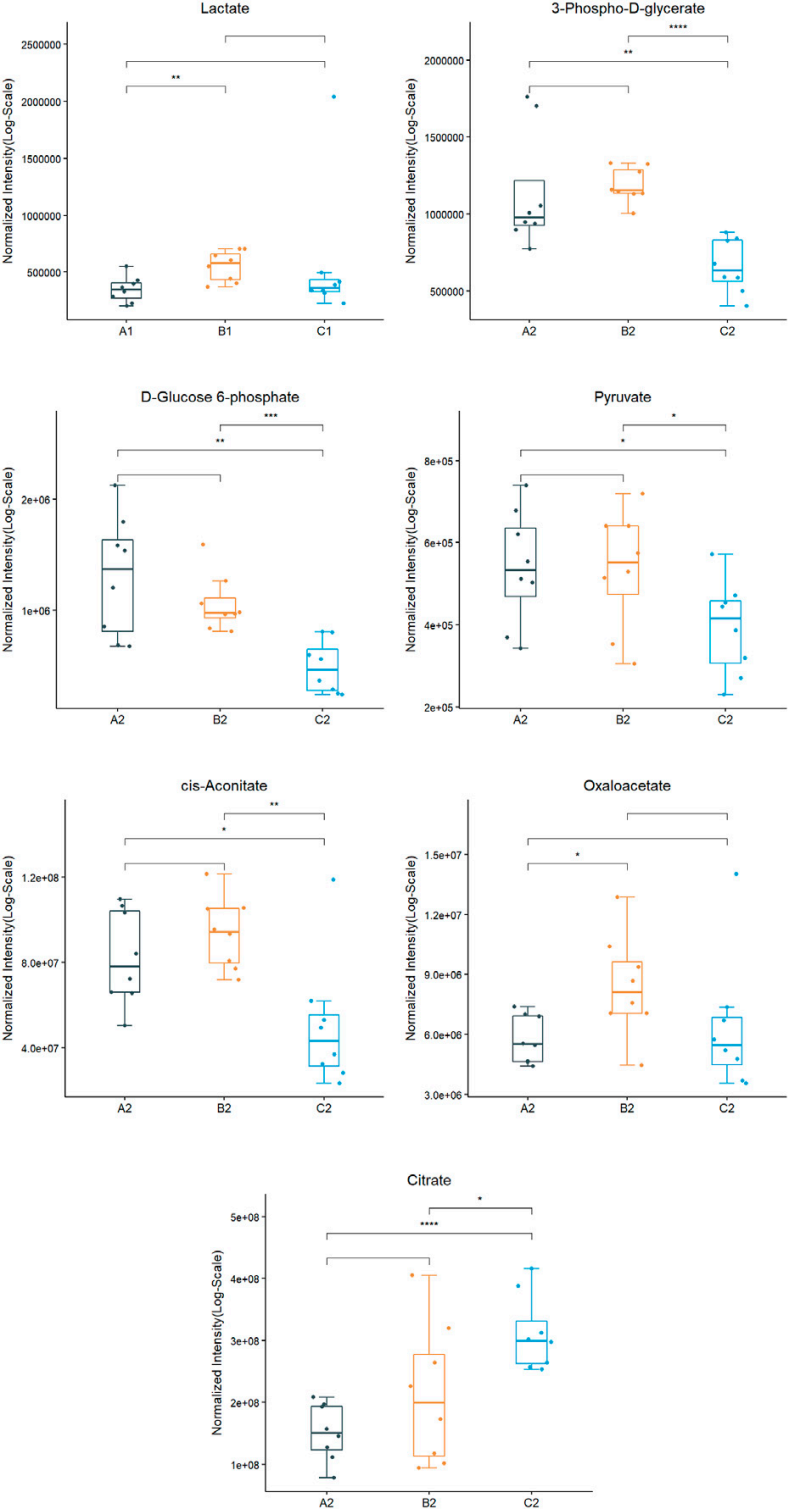
Metabolite expression trends between three experimental conditions. (A1: PL pre-exercise; B1: ES pre-exercise, C1: GS pre-exercise. A2: PL post-exercise; B2: ES post-exercise, C2: GS post-exercise.) * indicates *p* < 0.05; ** indicates *p* < 0.01; *** indicates *p* < 0.001.

There were five metabolic differences between the three experimental conditions at 50 min after the third WACT (see Figure 7). ES exhibited greater increases in 3-phospho-d-glycerate (*p* < 0.001), d-Glucose 6-phosphate (*p* < 0.001), pyruvate (*p* < 0.05), cis-aconitate (*p* < 0.01) than GS. The levels of 3-phospho-d-glycerate (*p* < 0.01), d-Glucose 6-phosphate (*p* < 0.01), pyruvate (*p* < 0.05), and cis-aconitate (*p* < 0.05) in GS were significantly lower than that in PL, while the level of citrate in GS was significantly higher than that in ES (*p* < 0.05) and PL (*p* < 0.001). Compared to PL, the level of oxaloacetate (*p* < 0.05) was higher after exercise in ES.

## Discussion

The main findings of this analysis were that, compared to PL, acute ingestion of ES and GS did not significantly improve either peak power or mean power during the WACT, regardless of the number of tests performed. However, compared to placebo, the intake of ES and GS increased the values of peak and mean power during the third and fourth WACTs (Figure 2). In addition, some additional changes occurred in ES and GS, including a decrease in the average power drop range. Our data suggest that in the population studied, enteric-coated and general sodium bicarbonate may have some positive impact on the Wingate tests on anaerobic capacity. One possible explanation for this finding is that power is more related to glycolytic metabolism and is limited by H^+^ accumulation (Lancha Junior et al., 2015; Lopes-Silva et al., 2019). Therefore, the buffering action of sodium bicarbonate could be more effective at minimizing fatigue and increasing mean power during the test, particularly in later stages (Lopes-Silva et al., 2019). Based on the literature reviewed, the failure of alkalosis to increase Wingate performance may be explained by an insufficient time during the exercise to allow a significant difference in H^+^ ion efflux from the muscle fibers or an inability to generate a sufficient difference in H^+^ ion gradient to produce a difference between trials (Mainwood and Worsley-Brown, 1975; Linderman and Fahey, 1991; Marx et al., 2002; Forbes et al., 2005; Street et al., 2005). Overall, these results suggest that sodium bicarbonate may have an ergogenic effect on repeated WACT test performance (Grgic, 2022). The likely explanation is also that recovery between bouts of exercise is enhanced by enhanced buffering capacity. However, one study has also suggested that sodium bicarbonate ingestion results in significant shifts in the acid-base balance of the blood and has a small but non-significant effect on anaerobic power and capacity (Parry-Billings and MacLaren, 1986). Our data are similar to the results of the study, but there is no significant change. This may be due to the higher intake dose in these studies than in the study we designed, although our dose is also effective in some studies (Durkalec-Michalski et al., 2020).

The findings demonstrated that BLA was not significantly different among experimental conditions in all tests. From the overall trend, the BLA of participants in enteric-coated and general sodium bicarbonate decreased more rapidly during the decline of BLA. We speculated that enteric-coated and general sodium bicarbonate might promote lactate clearance after exercise, reducing lactate accumulation. Some studies have indicated that sodium bicarbonate can change the content of blood lactate (Hartono and Sukadiono, 2017; Rezaei et al., 2019). Evidence suggests that sodium bicarbonate promotes non-oxidative energy metabolism, as observed through greater muscle lactate production and muscle glycogen utilization during intermittent exercise performed in the severe-intensity domain (Percival et al., 2015). However, if lactate is not removed in time, it will be decomposed and converted into lactate and cause a large amount of H^+^ accumulation, resulting in muscle acidification, causing acidosis (Zinner et al., 2011). The increase in buffering capacity achieved by ingesting sodium bicarbonate before exercise has previously been well documented (Siegler et al., 2010). Although our data suggest no significant difference among three conditions in the BLA (*p* > 0.05), the supplementation of enteric-coated and general sodium bicarbonate positively impacted the lactate clearance trend after exercise.

Three participants experienced serious problematic GI effects during our three experiments after taking general sodium bicarbonate and failed to complete the entire experiment. Previous studies have suggested that general sodium bicarbonate can adversely affect GI comfort (Cameron et al., 2010; Carr et al., 2011; Saunders et al., 2014). The accumulation of CO_2_ in the stomach, resulting from supplementation with sodium bicarbonate, may cause bloating, nausea, vomiting, and abdominal pain (Kahle et al., 2013). The incidence and severity of these side-effects increase linearly with the dose of sodium bicarbonate ingested and should be considered in terms of their overall effect on performance (Cameron et al., 2010). In this study, gastrointestinal discomfort was shown to be significantly more prominent in the general sodium bicarbonate trial than the placebo and enteric-coated sodium bicarbonate, resulting in an increase in stomach cramping, stomach ache, and diarrhea in the participants in sports. There was a larger increase in symptoms that may have impacted on performance. Therefore, following general sodium bicarbonate supplementation, these side effects may negatively impact exercise performance. More recent evidence suggests that enteric-coated sodium bicarbonate is a possible strategy to minimize the likelihood and severity of these side effects (Hilton et al., 2020). Our results support this study.

At baseline, no differences were observed among the three trials for HR. As expected, exercise resulted in increases in HR; all measures returned toward baseline during recovery from exercise (Table 2). This is similar to the study suggesting no improvement in heart rate recovery after exercise following sodium bicarbonate supplementation (Wang et al., 2019).

Based on RPE data, ES or GS did not affect RPE during a series of WACTs. This result disagrees with those published previously (Swank and Robertson, 1989; Swank and Robertson, 2002) showing lower RPEs under alkalotic conditions relative to placebo. Similar results have been demonstrated in the other study, showing that RPE measured immediately after each WACT was not affected by sodium bicarbonate ingestion (Zabala et al., 2008). This may be due to the excessive exercise intensity and short intervals during each WACT, resulting in little subjective feeling.

**FIGURE 8:**
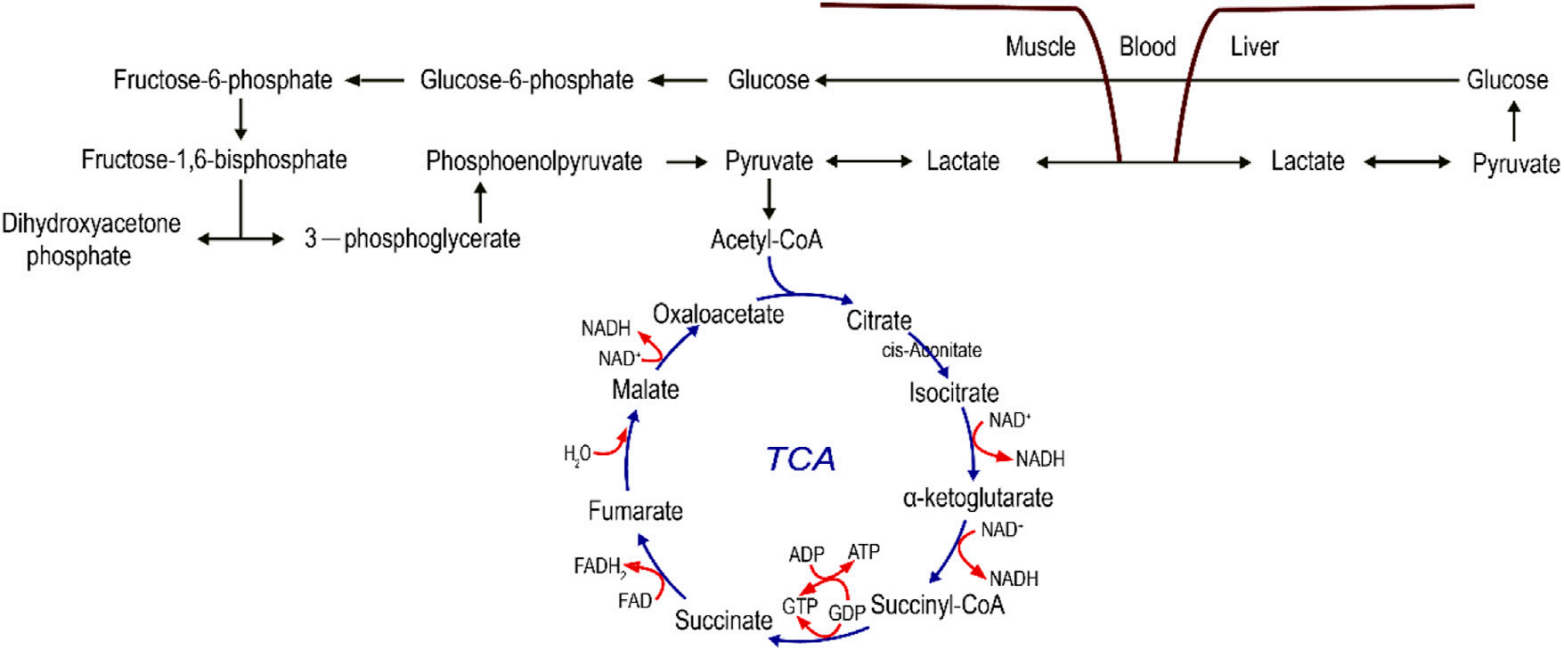
Pathways of related energy metabolites.

The targeted metabolomics tests cover important metabolites in the tricarboxylic acid cycle, glycolytic, and oxidative phosphorylation processes (see Figure 8). The metabolites of the participants in the three conditions were found to be diverse at that time. Studies have demonstrated that due to the high intensity and short movement duration, it is characterized by an energy supply derived primarily from glycolytic metabolism. In the ES, excluding citrate, the levels of 3-phospho-d-glycerate, pyruvate, cis-aconitate, and oxaloacetate were increased 50 min after exercise compared with the levels of exercise in the PL and GS, while d-Glucose 6-phosphate decreased. d-Glucose 6-phosphate, 3-phospho-d-glycerate, and pyruvate are the intermediate products of glucose catabolism. Glucose is the main energy-supplying substance in humans. ES more potently induced circulating glucose metabolism and the TCA cycle than GS and PL. It appears that both greater muscle buffering capacity and enhanced removal of protons result in increased glycolytic ATP production (Chycki et al., 2018). Citrate, oxaloacetate, and cis-aconitate are the intermediate products of the TCA cycle. The tricarboxylic acid cycle (TAC) is a necessary metabolic pathway in tissue energy supply and is distributed in the mitochondria. From the results, it is clear that the ES and GS affect the metabolic substances of the TAC metabolic pathway. Compared with the PL, an increase in intermediates of the TCA cycle was observed in the ES, including cis-aconitate, oxaloacetate, and citrate. It is hypothesized that the increased amount of TCA circulating intermediate in the blood may be due to the positive effect of ES intake on acidic substances clearance, which can promote the production of more energy in mitochondria for consumption by the system. The results showed that the differential metabolites related to the tricarboxylic acid cycle, aconitate and oxaloacetate decreased significantly, and citrate increased significantly, indicating the disorder of the tricarboxylic acid cycle. It may be due to side effects affecting the state of the exercise.

Lactate is a metabolite produced by the anaerobic enzymatic supply of glycogen and glucose outside the mitochondria in a state of oxygen deprivation. Various studies have confirmed that sodium bicarbonate increases extracellular pH and promotes H^+^ and lactate efflux from active muscles (Driller et al., 2012; Rezaei et al., 2019). The level of lactate in the ES of the pro-test was higher than that in the PL. It is hypothesized that ES supplementation promotes lactate clearance.

There are some limitations to the current study. Our study employed a limited sample of solely healthy young males, limiting its generalizability to other demographics. Coaches and athletes may consider using these supplements to give a possible performance edge, taking into personal account response.

## Conclusion

Based on these results, it was concluded that acute enteric-coated sodium bicarbonate and general sodium bicarbonate do not improve anaerobic exercise performance. Based on the analysis of target metabolomics, enteric-coated sodium bicarbonate and general sodium bicarbonate supplementation can affect the metabolism of substances, including 3-phospho-d-glycerate, d-Glucose 6-phosphate, pyruvate, cis-aconitate, oxaloacetate, citrate, and lactate. In healthy young men, the supplementation of acute enteric-coated sodium bicarbonate and general sodium bicarbonate can enhance some of the weak effects of blood lactate clearance during anaerobic exercise, which may be beneficial for glycolytic energy supply. An important point to note is that general sodium bicarbonate is a serious gastrointestinal problem, while enteric-coated sodium bicarbonate supplements can relieve gastrointestinal distress.

## Data Availability

The raw data supporting the conclusion of this article will be made available by the authors, without undue reservation.
